# ReMeDy: a platform for integrating and sharing published stem cell research data with a focus on iPSC trials

**DOI:** 10.1093/database/baab038

**Published:** 2021-06-22

**Authors:** Kirill Borziak, Irena Parvanova, Joseph Finkelstein

**Affiliations:** Center for Biomedical and Population Health Informatics, Icahn School of Medicine at Mount Sinai, 1425 Madison Avenue, Icahn L2-36, New York, NY 10029, USA; Center for Biomedical and Population Health Informatics, Icahn School of Medicine at Mount Sinai, 1425 Madison Avenue, Icahn L2-36, New York, NY 10029, USA; Center for Biomedical and Population Health Informatics, Icahn School of Medicine at Mount Sinai, 1425 Madison Avenue, Icahn L2-36, New York, NY 10029, USA

## Abstract

**Abstract:**

Recent regenerative medicine studies have emphasized the need for increased standardization, harmonization and sharing of information related to stem cell product characterization, to help drive these innovative interventions toward public availability and to increase collaboration in the scientific community. Although numerous attempts and numerous databases have been made to manage these data, a platform that incorporates all the heterogeneous data collected from stem cell projects into a harmonized project-based framework is still lacking. The aim of the database, which is described in this study, is to provide an intelligent informatics solution that integrates comprehensive characterization of diverse stem cell product characteristics with research subject and project outcome information. In the resulting platform, heterogeneous data are validated using predefined ontologies and stored in a relational database, to ensure data quality and ease of access. Testing was performed using 51 published, publically available induced pluripotent stem cell projects conducted in clinical, preclinical and *in-vitro* evaluations. Future aims of this project include further increasing the database size to include all published stem cell trials and develop additional data visualization tools to improve usability. Our testing demonstrated the robustness of the proposed platform, by seamlessly harmonizing diverse common data elements, and the potential of this platform for driving knowledge generation from the aggregation and harmonization of these diverse data.

**Database URL:**

https://remedy.mssm.edu/

## Introduction

Regenerative medicine is a promising therapeutic field, which aims at treatment, repair and replacement of injured cells, tissues and organs due to physical damages or degenerative diseases with healthy ones via various mechanisms. This innovative research includes stem cell therapies advancements, such as induced pluripotent stem cell (iPSC) treatments, that could potentially lead to the successful cure of currently incurable medical conditions by repairing the injury locally or replacing the damaged structure with a healthy transplantation product. In our previous work, we have discussed the significance of stem cell research, voluminous amount of available stem cell data and existence of many publically available stem cell data bases (1). Currently, the existing stem cell data are not consolidated, stored and available for access by researchers in a centralized and unified manner. Based on the necessity for stem cell data to be homogenously organized, deposited and visualized, we created Regenerative Medicine Data Repository (ReMeDy) platform (2), which can be publically accessed at https://remedy.mssm.edu/. ReMeDy is a unique repository, which allows the systematical collection and sharing of *in-vitro* findings and pre-clinical and clinical trial outcomes by using multi-modal common data elements (CDEs) framework, designed to include an essential set of CDEs, allowing the detailed comparisons across studies.

During the process of establishing the platform, we tested its functionality and usability by uploading 94 multi-modal CDE templates, based on 51 published, publicly available regenerative medicine CDE frameworks for *in vitro*, pre-clinical and clinical studies, indexed in the PubMed database. The functionality of the platform allowed each project to be successfully stored and accessed by utilizing visualization tools, project links and the application programming interface (API) platforms. ReMeDy platform is a user-friendly tool that allows effortless access to publically available studies, allowing the user to search by any CDE of interest across the uploaded studies.

## Materials and methods

### Database architecture and web interface

Our platform, called ReMeDy (2), is an implementation of the Signature Commons, a BD2K-LINCS (3) DCIC platform implemented through Docker and designed to store and search diverse metadata in an agile and flexible manner (3). The ReMeDy platform is installed on a Linux server using the default installation instructions. The Signature Commons platform (https://github.com/MaayanLab/signature-commons), on which ReMeDy is based, is composed of six repositories that are installed together through the Docker platform. These are the controller, data-api, metadata-api, proxy, schema and user interface (UI).

### Data types, literature search and data collection

Data for ReMeDy pilot-testing, in the format of the multi-modular CDE framework (1, 2), described below, were obtained from 51 published iPSC projects (Supplementary Table S1). The following articles were used as the dataset for this publication, identified by PubMed ID: 32632153 (4), 30651323 (5), 32929265 (6), 32165680 (7), 25436769 (8), 30119058 (9), 24020696 (10), 31227956 (11), 3253308 (12), 31547869 (13), 30691596 (14), 21303266 (15), 26971680 (16), 25143363 (17), 23515118 (18), 30912838 (19), 30535854 (20), 31445043 (21), 16308009 (22), 24006477 (23), 27075820 (24), 28073086 (25), 30582453 (26), 30772682 (27), 12084934 (28), 32353897 (29), 33154509 (30), 33142253 (31), 33137106 (32), 33130306 (33), 33108355 (34), 30738321 (35), 28296613 (36), 30224709 (37), 30449714 (38), 31577946 (39), 22495829 (40), 25479750 (41), 22895806 (42), 26494780 (43), 27099175 (44), 28282420 (45), 28436968 (46), 30876823 (47), 31107605 (48), 30442180 (49), 29800782 (50), 31373366 (51), 28967890 (52), 30712489 (53), 23029008 (54).

The template population process included the creation of a template for each stem cell product examined in each of the published projects included in our initial test set. Further, a template was created for each individual or grouped study subject (patient, animal model or cell line) reported in the set of publications, linking them to the stem cell product templates. The templates were then converted to JSON format and submitted for ingestion into the database using a custom Python script. Validation, visualization and user interface schema were similarly ingested. Specifically, a set of counting schemas was developed from the CDE framework to provide additional counting and filtering functionality to the search results page. These JSON format schemas, utilizing built-in Signature Commons functionality, were generated and ingested using a custom Python script. To further improve usability, the upload process was further improved by creating an upload interface using a front-end ReactJS and back-end Spring Boot. This upload interface allows for uploading and ingestion of CDE templates with minimal command line interface, while maintaining all of the validation features of the default ingestion pipeline.

## Results

ReMeDy is designed to be a user-friendly database, providing comprehensive and detailed information on induced iPSC projects. Given the public availability of all data contained within ReMeDy, it is freely accessible with no password or registration required. The search functionality and a tally of the projects contained within ReMeDy is accessible through the landing page. All projects contained within ReMeDy can be accessed through the Projects page. Additionally, the landing page provides access to the fully functional API interface. All web pages contain links to the Search, Project and API pages. This can help users find the needed function quickly. Users, who want to ask questions or provide a feedback, can find the links available at the bottom of the landing page. We plan to continuously update the database as new iPSC projects are published, in order to provide a useful and up-to-date resource to facilitate stem cell research and further driving knowledge generation.

ReMeDy is able to provide detailed information not only on the characteristics of iPSCs and their derivatives, similar to other stem cell databases, but, additionally, also on CDEs characterizing their derivation procedures. Additionally, ReMeDy provides CDEs characterizing the patients, animal models and cell lines under investigation in iPSC studies and their research findings. This new data resource has the potential to provide a unique opportunity to generate novel insights into the current state of iPSC research. By accessing this wealth of information in a harmonized, structured database, we aim to enable other researchers to gain a better understanding of the current landscape of iPSC and regenerative medicine research, provide insight into the best practices for generating iPSC and differentiated cell lines and provide information to help foster collaboration.

### ReMeDy platform

ReMeDy is an implementation of the Signature Commons, a BD2K-LINCS DCIC platform implemented through Docker, and is designed to store and search diverse metadata in an agile and flexible manner. The ReMeDy platform uses a relational database for data storage. Relational databases such as PostgreSQL, which is utilized by ReMeDy, excel at storing and searching structured data in a well-defined schema. While this comes at the expense of having to define the data structure ahead of time, it allows easy updating and indexing and very fast searches. Relational databases also enforce constraints based on the definition of primary and foreign keys and thus ensure the integrity of the data. With the large variety of data and metadata stored in ReMeDy in the form of CDEs and CDE values, PostegreSQL represents a means to cleanly manage structured data such as small molecules, cells and genes, without compromising performance of searching very large data sets, by storing the sets as key value pairs. Furthermore, the leveraged data technology alongside a properly implemented schema for data storage provides strong data conformity to the FAIR guidelines (findable, accessible, interoperable and reusable). Additionally, the PostgreSQL database has tables for each class of object and defined relationships with strong restrictions preventing erroneous updates. Indexing enables very fast searching for nearly any attribute of the metadata without major slowdowns as the size of the tables expand.

**Figure 1.**
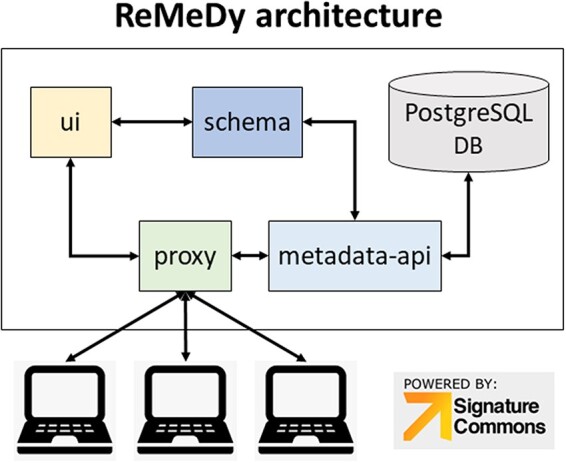
ReMeDy platform architecture that is based on an implementation of the NIH BD2K-LINCS DDCIC developed Signature Commons platform, installed through Docker. The figure illustrates the interaction between the Docker packages and the PostgreSQL database.

The Signature Commons platform, on which ReMeDy is based, is composed of six repositories that are installed together through the Docker platform. These are the controller, data-api, metadata-api, proxy, schema and UI. The main functions of the database, are done by the proxy, metadata-api and UI containers (Figure 1). The proxy repository is used as the primary access point to the ReMeDy platform and provides the coordination between the UI and API services. It is a convenient and generic proxy image. It is useful for exposing multiple microservices on a single ingress and easily configurable via environment variables. The UI repository is the front-end UI for displaying the API integration. This repository is responsible for the visualization of stored data, the search functionalities and graphical representation tools. The metadata API repository, powered by LoopBack, is responsible for communication with the PostgresDB, which is used to store the CDE data and functions as both to retrieve and upload data. The controller repository is used as an intermediary to aid the data ingestion process. It is responsible for preparing and controlling ReMeDy with existing data/store. This repository includes scripts to facilitate data ingestion from different forms to those that can be used by the signature commons. It can subsequently upload the processed data to the Signature Commons through the relevant APIs. The data-api repository is responsible for the set up and installation of the ReMeDy instance. It is built using Gradle, a build automation tool for multi-language software development. It controls the development process in the tasks of compilation and packaging to testing, deployment and publishing. The function of data-api is to compile production Java source files using the JDK compiler, deploy Web Application Resource (WAR) files, start a Tomcat instance and deploys the WAR, aggregate task that performs verification tasks such as running the tests and run the unit tests using JUnit or TestNG. Born out of the Apache Jakarta Project, Tomcat is an application server designed to execute Java servlets and render web pages that use Java Server page coding. The schema package contains information on the JSON-Schema validators for ReMeDy entities, designed to allow one to flexibly validate arbitrary metadata in the ReMeDy database.

**Figure 2.**
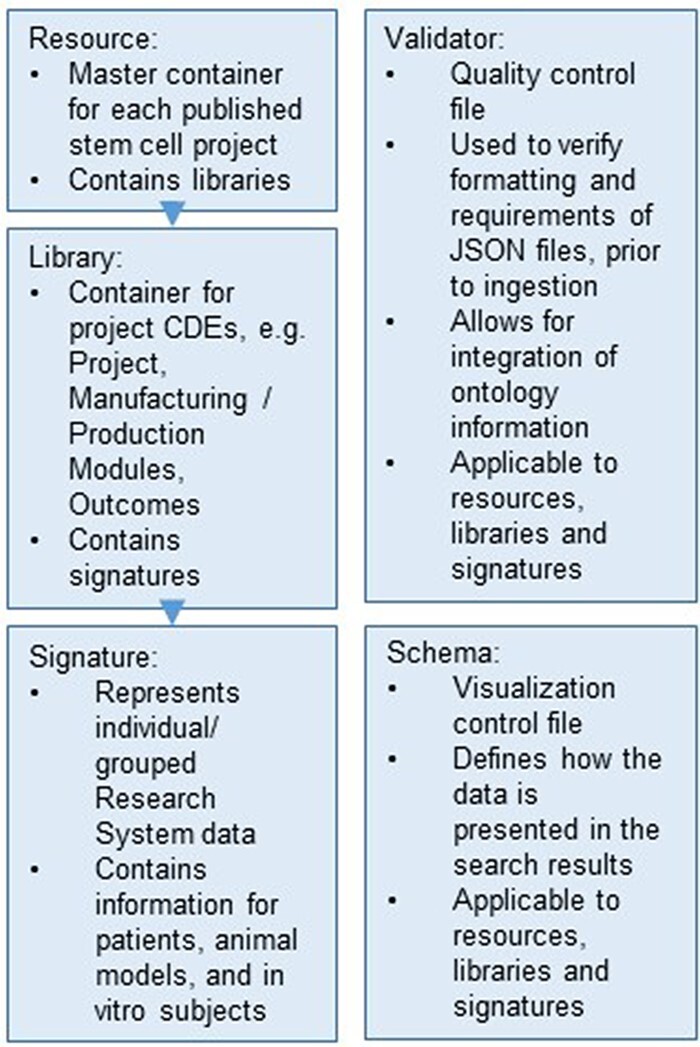
Hierarchical organization of the data stored within the ReMeDy platform.

The database structure of ReMeDy is based on Signature Commons, which is organized into four containers: resources, libraries, signatures and schemas (Figure 2). Each of the containers is references by its own RESTful API endpoint. All of the data entities within each of containers is referenced by a universally unique identifier (UUID) to ensure data integrity and interoperability. Resources are the top-level containers. In the context of iPSC projects, they represent individual publications, from which information related to iPSCs, stem cell products and research subjects are linked. Libraries link to resources using UUIDs and act as the intermediate level data storage entities. Libraries serve to store the majority of information about the iPSCs, which includes manufacturing and production, donor information and information about the in-depth product characterization assays (omics). Libraries additionally act to store clinical outcomes and research findings CDEs. Libraries, in turn, link to signatures. In ReMeDy, signatures represent individual or grouped research subjects. Research subjects include patients, animal models and *in-vitro* cell lines. Signatures contain CDEs that encode baseline and follow-up data and CDEs that encode assays that were used to characterize the research subjects. The schemas container contains JSON entities that are used to modify the display information of the UI, resources, libraries, signatures and are used to design the filtering schemas for improving the ReMeDy search interface.

The final data type is the validator, which represents the quality-control measure to ensure appropriate data structure and integrity. These JSON files contain instructions of what different elements of the resources, libraries, signatures and schemas must contain in order for them to be acceptable for ingestion into the database. Validators serve as the method for verifying the integrity of CDE values against external ontologies, such as UniProt and NCBI Gene. Due to their critical role in aiding data ingestion, validators are not stored within the PostgreSQL database and are instead installed as part of the schemas package. The data within each entity are in turn hierarchically organized. Resources, libraries, signatures and schemas contain a minimal set of elements that are required to ensure a proper data structure. These include UUIDs, UUID links to libraries and signatures and links to the appropriate validators for each entity type. The majority of data from each entity is stored within the meta-element. The meta-element also contains a link to a metadata validator, which enables for directed validation of data against external ontologies. This nested organization enables ReMeDy to take metadata of varying structure while ensuring the integrity of the key data structures.

### ReMeDy published iPSC project dataset

Results from numerous published clinical and pre-clinical iPSC-based studies were used to test the functionality of the platform (Supplementary Table S1). Here, we show the 51 studies that were used as part of the test dataset. In the current format, ReMeDy contains over ∼70 (average used per study)/820 (in total) CDE values, with a core set of over 20 CDEs common to all projects. For the ReMeDy implementation, resources represent the individual research projects. Each project has at least one library, which contains the static CDEs, i.e. those that are not expected to change across the time course of the project, such as project information, cell product manufacturing and production information and in-depth product characterization. In essence, each library represents each unique iPSC formulation used in each trial, particularly containing the detailed information on the production and derivation of the iPSC-based stem cell product. Further information on the structure and organization of the CDEs contained in the libraries is provided below in the Modular CDE Framework section. The libraries, in turn, contain signatures. Signatures represent individual or grouped study subject and animal model records and contain demographic, baseline, outcome and adverse event CDEs. As individual research subject results are often not included in the published results, we used group averaged results as available to represent the outcome information of the iPSC-derived stem cell products on the research subjects. This hierarchical approach allows us to capture all the relevant information with regard to the iPSC products related in each publication, in a format that is easily queried, visualized and intuitively organized to improve user engagement.

**Figure 3.**
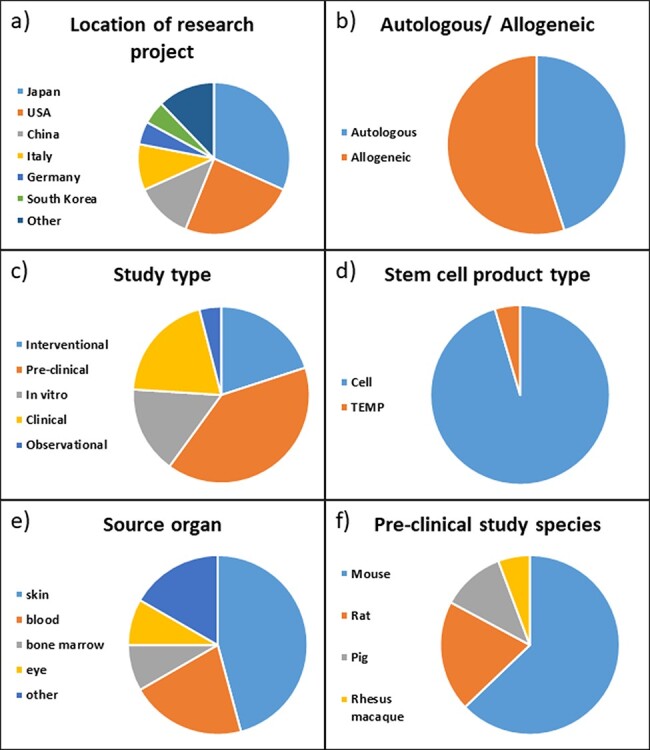
Distribution of projects in ReMeDy database by (a) location of the rearch project, (b) autologous or allogeneic treatment type, (c) study type, (d) stem cell product type, (e) source organ for iPSCs and (f) pre-clinical study species.

To test the functionality and improve the usability of the ReMeDy platform, we strived to include a diverse set of published projects (Figure 3). The first interesting feature of the studies in our trial dataset is that the majority of them were conducted in either Japan or the USA, 32% and 24%, respectively (Figure 3a). Other active contributors to stem cell research appear to be China and Italy, with 12% and 10%, respectively. Other countries with published stem cell research in our dataset were Germany, South Korea, Sweden, Argentina, Saudi Arabia, the UK and Pakistan.

The projects were nearly evenly split between autologous and allogeneic treatment types, with 45% and 55%, respectively (Figure 3b). Just under half, 44%, of the included studies were performed on patients, this includes 20% of studies designated as clinical, 20% designated as intervention and 4% as observational (Figure 3c). A further 40% were pre-clinical studies performed on animal models, with the remaining 16% being *in-vitro* studies that focused on the derivation of iPSCs or their differentiated products. The vast majority of the studies, 95%, focused specifically on cell products, while only 5% used a tissue engineered medical product (TEMP) in their research (Figure 3d). The source organ for the majority of derived stem cells was skin, being utilized in 39% of the studies (Figure 3e). The skin fibroblast cell lineage was the most common skin cell type to be reprogrammed into iPSCs. Blood was the second most common with 18% of studies utilizing it. Other source organs for iPSCs in our set of published studies included bone marrow, eye, lung, iliac crest and umbilical cord. Of the pre-clinical studies, the vast majority, 63%, were performed on mice. A further 20% of the studies were performed on rats, 11% on pigs and 6% on rhesus macaques (Figure 3f).

The published studies focused on a wide range of disease conditions, with 15 different conditions documented in the database. The most common disease condition under investigation in the set of published studies in ReMeDy was cancer, which accounted for 14% of the studies. Other conditions documented include age-related macular degeneration, hamyotrophic lateral sclerosis, aplastic anaemia complicated by platelet transfusion refractoriness, end-stage respiratory malfunctions, Gaucher disease, graft versus host disease, hereditary pulmonary alveolar proteinosis, hypertension, inherited erythromelalgia, ischemic cardiomyopathy, ischemic stroke, multiple sclerosis, myocardial infarction, neurodegenerative disorders, neurofibromatosis type 1, Parkinson’s disease, retinitis pigmentosa, short QT syndrome, spinal cord injury, spinal muscular atrophy with respiratory distress type 1, stroke, traumatic brain injury and type IV congenital dyserythropoietic anaemia.

### Data upload interface

**Figure 4.**
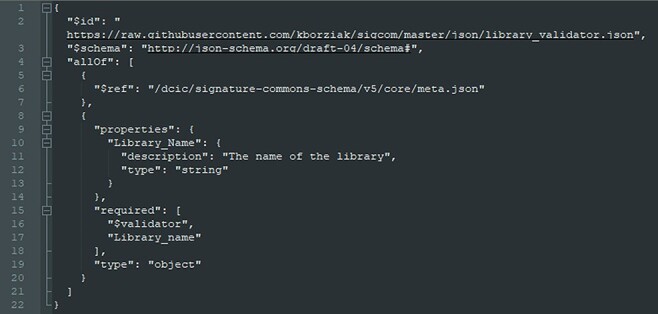
Example JSON format file used for interface with ReMeDy API.

CDE data are ingested into and downloaded from the ReMeDy platform using the fully functioning API, through JSON format files (Figure 4). The API is annotated using Swagger 2.0 JSON implementation, and all RESTful endpoints return structured JSON. Multiple endpoints allow searching content by any of its curated metadata annotations as well as requesting different slices of metadata associated with any of the subcategories of CDE modules.

To further improve the usability of ReMeDy, we implemented an interface to allow quick and efficient data upload by researchers that builds upon the core RESTful API available through Signature Commons. The front end of the interface is built on ReactJS, while the back end of the interface was created using Spring Boot. The advantage of this interface is its ability to generate JSON using only an input tab delimited file, generated from the CDE template, while adding and incorporating required features such as generating UUIDs. The upload interface allows for creation of all three data types: resources, libraries and signatures. Further, addition of progress bars allow the user to visualize the upload of the data, JSON generation and API submission. Following successful upload of the data, the back-end application reads the file using ‘tsvReader’ API. Next, the back end generates JSON file with the pre-defined type and content, by converting every entry of uploaded file into a ‘JSON Object’. The resultant JSON String is submitted to the database using the Signature Commons API.

Due to the hierarchical nature of our data, the CDEs need to be ingested procedurally. Since signatures require UUID links to a library and libraries require UUID links to a resource, first a resource has to be created for each funded project. Next, the library JSON files are generated, containing the CDEs that are static across all study subjects of each project. Finally, signature (patient, animal model or cell line, as appropriate) JSON files are generated and linked to the relevant libraries. For the library and signature JSON files in this study, custom metadata validator JSON files were generated (Figure 2). These metadata validators define which key value pair elements the metadata will contain, the format of the values (including validation against ontologies), and identify required elements for ingestion. This quality control step allows for the final verification of CDE formatting prior to ingestion into the system. Following successful ingestion, schema JSON files were ingested into the ReMeDy. These files define the visual presentation of the libraries and signatures within the ReMeDy search results, allowing us to define the most informative CDEs to present the viewer in aiding their efforts to further refine their search results.

### Multi-modular CDE Framework

In order to facilitate data collection and promote a standardized organization of data within the database, we developed a multi-modular CDE framework (Supplementary Table S2), which aims to capture all facets of information related to iPSC projects. Previous attempts to create standardized frameworks for characterization of stem cells have resulted in the creation of the Minimum Information About a Cellular Assay for Regenerative Medicine (MIACARM) (55), a format in the process of being adopted by major stem cell banks such as hPSCreg. While MIACARM creates a functional organization of CDEs related to stem cell characteristics, it does not provide any CDEs to cover the other areas of data generated by iPSC projects needed to gain a full perspective of the work being done, such as research subject and project outcome information. Our multi-modular CDE framework aims to address these deficiencies by using a scoping review approach for defining relevant CDEs (1).

**Figure 5.**
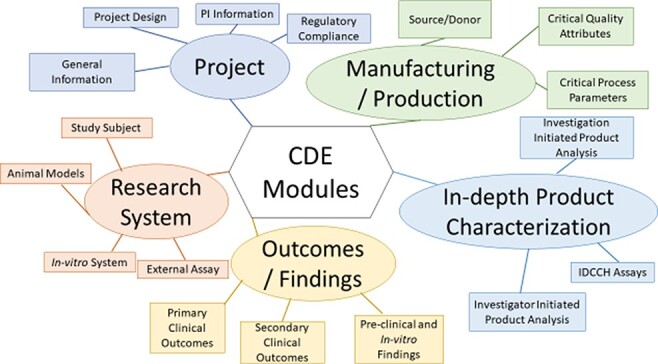
Organization of the Multi-modular CDE Framework used for extracting data from published iPSC projects.

The framework consists of five modules, which are Project (containing pertinent project status and Principal Investigator (PI) CDEs), Manufacturing/Production (containing donor, Critical Quality Attributes and Critical Process Parameters CDEs used for generating iPSCs and cell products derived from them), In-depth Product Characterization, Research System (containing patient, animal model and cell line CDEs) and Outcomes/Findings (Figure 5). The CDEs within the modules are further hierarchically organized into sections and subsection, to allow for easy navigation of the framework (Table 1). An example subset CDEs, detailing the hierarchical organization from module to individual CDEs for the Donor Information subsection of the Manufacturing/Production Module, of the multi-modular CDE framework is provided (Table 2). This table illustrates the five CDE modules, the sections of the Manufacturing/Production module, the subsections of the Source/Donor section, the CDEs contained within the Donor information subsection and the detailed descriptive information provided for each CDE. This includes the CDE name, a CDE description, permissible values/ontology information used to standardize and harmonize CDE values and an applicable project-type guide, which delineates the utility of the CDE to either clinical, preclinical or *in-vitro* studies. A full version of the CDE framework can be found in Supplementary Table S2.

**Table 1.** Table describing CDE organization and content across the five modules of the multi-modular CDE framework

Module	Section	Subsection	CDE content
Project	General Information		Project descriptive information such as project title and key words
		Investigational New Drug Application (IND)/Investigational Device Exemption (IDE) Information	FDA defined IND and IDE descriptive CDEs
	PI Information		General PI information
		Contact Information	Detailed PI contact information
		Physical Address	CDEs specific to physical location of the study
		Project Contact Roles	Names of other collaborators
	Publication/reference information		CDEs characterizing the publication
	Project Design		General project design information, such as trial phase and inclusion/exclusion criteria
		Study Design	Specific study design data such as participant allocation
		Study Population	Specific information about the study population
		Administrative Practice	CDEs characterizing the administration of the stem cell products
	Regulatory compliance		Information about regulation of the project
		Study Monitoring	Study monitoring information
		Individual Participant Data Sharing Statement	CDEs describing sharing of participant data
Research System			General research subject information, such as subject ID
	Study Subject	Informed Consent	Patient consent CDEs
		Demographics	Patient demographic information
		Physical/Medical Status	Patient health information
		Socioeconomic Status	Patient socioeconomic information
		Lifestyle	Patient lifestyle information, such as smoking status
		Environmental conditions	Patient living conditions
	Animal Model	Animal Characteristics	General animal model characteristics such as species and age
		Animal source	Source of the animal models
		Species and strains	Detailed species and strain CDEs describing animal model
		Sex and related information	Sex information of animal model
		Genetic modification	Genetic characteristics of animal model
		Housing	Housing information
		Diet	Diet information
	In-vitro System	Identification	Cell line identification information
		Origin	Origin information of the *in-vitro* system
		Cell line properties	Properties of the cell line, such as growth and morphology CDEs
		Propagation	CDEs describing the propagation of the cell line
	Experimental Assays	Assays	General assay descriptive CDEs
		Assay metadata	Detailed assay description, including data type and processing CDEs
		Imaging	CDEs describing imaging data
		Transcriptome Profiling	CDEs describing transcriptomic profiling of research subjects
		Genotype Profiling	CDEs describing genomic profiling of research subjects
		Genetic engineering	CDEs specific to genetic modification of research subjects
Manufacturing/Production			General information such as stem cell product ID
	Source/Donor	Donor information	General donor information, such as species and age
		Anatomical origin	Anatomical origin of source cells
		Source cell information	Detailed information on specific source cells transformed into iPSCs
	Critical Quality Attributes	Product Type	General descriptive CDEs of stem cell product
		Sterility	CDEs describing sterility of stem cell product
		Release Criteria	Specific criteria critical for final stem cell product quality assurance, such as cell morphology and cell potency
	Critical Process Parameters	Manufacturing Information	Manufacturing information of the stem cell product, including passage number and scaffold information
		Preparation of Cellular Component	General descriptors of cell culture and reprogramming conditions
		Sample Storage	CDEs describing sample storage
		Materials used for culture of source cells	Descriptors of source cell culturing
		Materials used for source cell procurement	Descriptors of source cell procurement, such as container information
		Materials used for culture in stem cell establishment step	Descriptors of culture materials used in establishment of the stem cells
		Materials used for culture in final step of stem cell production	Descriptors of culture materials used in preparation of final stem cell product
		Materials used for stem cell storage and transfer/shipping	CDEs describing stem cell storage
		Cell banking process: Source cell procurement process	CDEs describing transferring of source cells
		Cell banking process: QC for stem cell storage/banking conditions	CDEs describing banking of stem cells
In-depth Product Characterization	IDCCH Assays	Microbiology/Sterility Testing	Descriptors of bacterial and fungal sterility testing
		Pyrogenicity/Endotoxin Testing	Descriptors of endotoxin testing
		Virology/Adventitious Agents	Descriptors of virology testing
		Mycoplasma Testing	Descriptors of mycoplasma testing
		Identity (Species determination) and Sex (Male/Female)	Descriptors of species and sex verification
		Purity and Heterogeneity	Descriptors of product purity
		Viability	Descriptors cell viability
		Senescence	Descriptors cell senescence
		Genetic Stability	Descriptors of product genetic stability
		Cell Proliferation	Descriptors of cell proliferation, including for Luminescence ATP assay
		Clonal Capacity	Descriptors of product clonal capacity
		Pluripotency/Multipotency	Descriptors of product pluripotency
		Tumorigenicity	Descriptors of product tumorigenicity
		Transcriptome Profiling	Descriptors of product transcriptomic profiling
		Epigenome	Descriptors of product epigenomic characteristics, such as ATAC-seq and ChIP-seq assays
		Genome Profiling	Descriptors of product genomic profiling
		Proteome	Descriptors of product proteomic characterization, including instrument characteristics
		Lipidome and Metabolome	Descriptors of product lipidome and metabolome characterization
		Comprehensive Assessment of Cellular Markers	Descriptors of product cellular marker assessment, such as Flow cytometry analysis
		Potency	Descriptors of product potency, such as Cytokine secretion and Electrical resistance analysis
	Investigation Initiated Product Analysis	Assays	General information on stem cell product assays
		Assay metadata	Detailed descriptors of data generation during assays, such as data processing and normalization methods
		Imaging	CDEs describing imaging data
Outcomes/Findings	Primary Clinical Outcomes		Description of primary clinical outcome
	Secondary Clinical Outcomes		Description of secondary clinical outcomes
	Preclinical and In-vitro Findings		Description of experimental findings from preclinical and *in-vitro* findings

The table columns module, section and subsection describe the hierarchical organization of CDEs within the multi-modular framework. Modules contain sections, which in turn contain subsections. The CDE content column describes which CDEs each subsection and section, for those CDEs not assigned to a subsection, contain.

#### Project module

The Project module contains CDEs that capture information about the PI, general project summary information, such as grant number, project design and regulatory compliance. Aside from the general project information, which includes CDEs for project title, description, key words, hypothesis and start and end dates, as well as FDA-regulated Investigational New Drug (IND) Application/Investigational Device Exemption (IDE) information, the module contains four additional sections: PI Information, Publication/reference information, Project Design and Regulatory Compliance. The PI Information section contains CDEs for PI Name and Primary Clinical Study Sponsor, as well as three subsections: Contact Information, Physical Address and Contact Roles. Contact Information contains CDEs that describe the contact information for the PI, including email, physical address and the academic degrees of the PI. The Physical Address subsection has CDEs that describe the physical address of the trial site. Specifically, these CDEs characterize the trial site location from the country level down to a specific street address. Finally, the Project Contact Roles subsection contains CDEs that provide names of additional research members, such as biostatistician, medical monitor and technical lead. The Publication/reference information section contains CDEs that describe the publication indexed in ReMeDy. This information includes publication title, authors, journal name, PubMed ID and a link to the referenced publication.

The Project Design section contains CDEs that characterize the design of the project, which includes CDEs such as trial phase, inclusion/exclusion criteria and target sample size. This section is further delineated into three subsections: Study Design, Study Population and Administrative Practice. The Study Design subsection contains CDEs that provide a detailed characterization of the study. These include Participant Allocation, Randomization Type, Stratification and Target Follow-Up Duration. The Study Population subsection describes in detail the parameters of the study population, with CDEs such as Comorbid Condition, Demographic Group, Population Rationale and General Health Status. Finally, the Administrative Practice subsection is devoted to CDEs that characterize how the stem cell products are administered to the patients or animal models. These CDEs characterize in detail the specifics of the administered product, such as cell count, route of administration, frequency of administration and the administration protocol. The Regulatory compliance section CDEs describe the study’s compliance with regulatory bodies such as Institutional Review Board and Data Monitoring Committee, along with status of obtaining consent and indicators of registering the trial with appropriate bodies such as ClinicalTrials.gov. This section is also further subdivided into two subsections: Study Monitoring and Individual Participant Data Sharing Statement. The Study Monitoring subsection contains CDEs that further detail the monitoring of clinical trials, such as risk assessment and monitoring, and whether the clinical trial is FDA regulated as defined in the U.S. Public Law 110-85, Title VIII, Section 801. The Individual Participant Data (IPD) Sharing Statement CDEs describe in detail the sharing plan and time frame for the IPD.

#### Research System module

The Research System module contains CDEs characterizing both patients and model organisms, designed to accommodate study patients, animal models and derived cell lines. Further, the CDEs contained here are designed to accommodate both baseline and treatment follow-up information. This module also contains CDEs to characterize any experimental assays the research subjects may undergo during the period of the trial, including transcriptomic profiling and genomic sequencing. In addition to general CDEs such as subject ID, administered product ID and subject data type (which describes if the reported subject data are from individuals or reported as grouped averages in the publication), the CDEs in this module are organized into four section: Study Subjects (which contains patient CDEs), Animal Model, In-vitro System and Experimental Assays. This organizational approach allows us to provide a focused data storage and retrieval system, which makes it easy to retrieve CDEs specific for patients versus animal models, for example. Further, this approach allows for focused data import, by allowing researchers to focus on CDEs that are relevant to their study type.

**Table 2.** Hierarchical nesting architecture of CDE framework with a detailed view of the CDEs contained in the donor information subsection

Module	Section	Subsection	CDE
Project			
Research System			
Manufacturing/Production	Source/Donor	Donor information	Donor organism
			Sex
			Age
			Race
			Ethnic group
			Donor genetic information
			Health/disease status when obtained
			Donor permission
		Anatomical origin	
		Source cell information	
	Critical Quality Attributes		
	Critical Process Parameters		
In-depth Product Characterization			
Outcomes/Findings			

The module column lists the five modules of the framework. The section column details the sections of the manufacturing/production module. The subsection column details the subsections of the source/donor section. The CDE column lists the CDEs that belong to the donor information subsection.

The Study Subject section patient CDEs are further subdivided into six subsections: Informed Consent, Demographics, Physical/Medical Status, Socioeconomic Status, Lifestyle and Environmental conditions. The Informed Consent subsection contains CDEs describing when informed consent was obtained, as well as the informed consent form and protocol version information. The Demographics subsection contains detailed information on the demographics of the patient, including sex, gender, age, race and ethnic group data. The Physical/Medical Status subsection contains the majority of disease and study-specific CDEs related to measuring the medical parameters of each patients. Among the CDEs currently in this subsection are those characterizing health or disease status, the specific disease diagnosis, risk assessment, height and body weight. The modular nature of the framework in combination with the flexible database structure of ReMeDy allows us to easily add additional disease or medical condition CDEs, as required by new published projects added to the database. Current disease-specific CDEs include those monitoring coronary artery calcium volume and calcification, blood parameters such as basophil count, blood pressure, cholesterol parameters, number of gadolinium-enhancing lesions, Expanded Disability Status Scale and T2 lesion volume. The Socioeconomic Status CDEs cover information related to employment status, marital status and education. The Lifestyle subsection CDEs cover information related to smoking status, drug and alcohol use, and diet. The Environmental conditions subsection CDEs characterize the environmental setting of the patient, with information such as urban/rural status, superfund area status and altitude.

The Animal Model section is designed to accommodate any animal model that can be used for iPSC research. As part of our database testing, we are able to accommodate information from a wide range of common animal models, including mice, rats, pigs and monkeys. The Animal Model section is subdivided into the Animal Characteristics, Animal source, Species and strains, Sex and related information, Genetic modification, Housing and Diet subsections. The Animal Characteristics subsection contains CDEs that detail the general animal characteristics such as animal species, sex and date of birth. Animal source CDEs detail the vendor of the animal models, their location, breeding scheme, facility arrival date and quarantine procedures for the animals. The Species and strains subsection contains detailed information of the strains of specific animal models, particularly the detailed identification of rat or mouse strains used in the study. Similarly, the Sex and related information subsection provided CDEs that additionally detail sex information such as gonadectomy or ovariectomy. The Genetic modification subsection CDEs prove details on the genetic changes to the animal model strains performed prior to the study experiments. This includes data on breeding scheme and transgenic modification, such as whether the transgenic modification is conditional, inducible, a deletion or a mutation. The Housing subsection provides CDEs that characterize information such as number of animals per cage, cage size and material, bedding, room temperature and lighting. The Diet submodule provides information on the vendor of the animal food, as well as the food and water delivery systems.

The In-Vitro System is used to characterize *in-vitro* cell line experiments and consists of four subsections: Identification, Origin, Cell line properties and Propagation. The Identification subsection contains CDEs that are used to identify individual cell lines, such as cell line name. The Origin subsection contains CDEs that describe the donor and tissue from which the cell line was originally obtained. Cell line properties CDEs describe growth rate, cell morphology and cellular products that define the cell line. The Propagation subsection details the conditions used to maintain the cell line, such as medium, atmosphere, seed density, split ration and detachment aids.

The Experimental Assays section, which details the experiments performed in the research system in order to test the stem cell products, is composed of six subsections: Assays, Assay metadata, Imaging, Transcriptome Profiling, Genotype Profiling and Genetic engineering. The Assays subsection CDEs describe the general descriptive characteristics of the assay types utilized in each project. The Assay metadata subsection CDEs detail the data types generated from the assays, as well as their processing, normalization and analysis methodology. The Imaging subsection contains CDEs that characterize collection of imaging data such as from MRI scans. The Transciptome Profiling subsection CDEs detail the experimental strategy, sequencing platform and workflow used to obtain transcriptomic information from the research subjects. Similarly, the Genotype Profiling subsection contains CDEs that detail how research subject genotype information is obtained. Finally, the Genetic engineering subsection CDEs describe in detail the type and location of genetic modifications that the research subjects have undergone as part of the stem cell product research. Similarly to the Physical/Medical Status subsection, we intend the Experimental Assays section to be expandable as additional projects are added to the ReMeDy database. This will allow us to add additional CDEs and module subsections to detail additional Experimental Assay, such as Proteomic or Epigenomic Assays, as they become utilized in these additional projects.

#### Manufacturing/Production module

The Manufacturing/Production module is designed to capture information about the stem cell product, including critical quality attributes and good manufacturing practices that will be crucial to ensure the standardization of the product to enable future collaborative sharing of scientific data. This module also contains the donor and source information for the stem cell product under investigation, including Master cell bank information. In addition to general descriptive CDEs such as Product ID, the Manufacturing/Production module is divided into three sections: Source/Donor, Critical Quality Attributes and Critical Process Parameters.

The Source/Donor section contains all CDEs used to identify and characterize the original source of the stem cell product. It is further subdivided into three subsections: Donor information, Anatomical origin and Source cell information. The Donor information subsection describes the species, age, sex, health and genetic information of the donor organism. Additionally, this section contains a CDE to characterize donor permissions for use or sharing of the source cells. The Anatomical origin subsection CDEs detail the specific anatomical location from which the source cells for the iPSCs were obtained, from the broadest organ category to the specific cell lineage of the source cells. The Source cell information subsection further describes the specific source cell line that was utilized for induction of pluripotency and subsequent experiments. CDEs in this subsection include source cell karyotype, blood group, HLA genotype, autologous versus allogeneic designation, as well as Master Cell Bank ID and providing institution name for projects where source cells were not obtained directly from the subject.

The Critical Quality Attributes section details the iPSC or iPSC-derived stem cell product attributes that are critical for specifically defining and delineating the product. This section is further subdivided into Product Type, Sterility and Release Criteria subsections. The Product Type subsection details the type of stem cell product under investigation. Although the majority of iPSC project focus specifically on the cells, we have included additional CDEs to designate if the product is a TEMP or is potentially an intervention product contain exogenous genetic material. As TEMPs are becoming more common, we have included TEMP projects in our initial set of ingested publications. The Sterility subsection contains CDEs that characterize sterility tests performed on the final stem cell product, including bacterial, fungal, mycoplasma, protozoal and viral tests. The Release Criteria subsections contain the majority of critical CDEs used to define the stem cell product and its characteristics. The CDEs define many critical parameters, including cell potency, cell viability, morphology, population purity, differentiation propensity and viral copy number, in addition to epigenetic, protein, cell surface and metabolite markers that are used to define the stem cell products under investigation. Finally, this subsection also includes CDEs that characterize critical scaffold parameters for TEMPs, such as scaffold thickness and minimum mechanical strength.

The Critical Process Parameters section contains CDEs that are used to characterize the process that was used to transform the source cells into iPSCs and the final stem cell product. These process parameter CDEs are subdivided into 10 subsections: Manufacturing Information, Preparation of Cellular Component, Sample Storage, Materials used for culture of source cells, Materials used for source cell procurement, Materials used for culture in stem cell establishment step, Materials used for culture in final step of stem cell production, Materials used for stem cell storage and transfer/shipping, Cell banking process: Source cell procurement process, and Cell banking process: QC for stem cell storage/banking conditions. The Manufacturing Information subsection contains CDEs that characterize the manufacturing of the final stem cell product, particularly for TEMPs. This includes CDEs for cell information of the final product, such as passage number, sterility and other in-process controls, and information on the scaffold material used to generate the TEMPs, such as scaffold composition and biodegradation properties. The Preparation of Cellular Component CDEs define the culture media, passage control and type of reprogramming the cell underwent to generate the final cellular component product. Sample Storage subsection CDEs describe the acceptability criteria for shipping and administration of the final stem cell product used in the published studies. The Materials used for culture of source cells subsection contains CDEs that define the conditions used to culture the source cells, including CDEs for basal medium, culture additives, antibiotics, feeder cells, basement membrane and sterility considerations. Similarly, the Materials used for culture in stem cell establishment step and Materials used for culture in final step of stem cell production subsections contain CDEs which describe the culture materials used during stem cell establishment and during differentiation of the stem cells into the final stem cell product. The Materials used for stem cell storage and transfer/shipping CDEs define which materials were used to store the iPSCs. The Cell banking process: Source cell procurement process subsection CDEs describe the source cell procurement, transferring and storage/banking protocols. Finally, the Cell banking process: QC for stem cell storage/banking conditions subsection defines the quality control criteria for stem cell storage, such as the cryopreservation method and temperature.

#### In-depth Product Characterization module

The In-depth Product Characterization module contains CDEs related to the assays used for the full standardized characterization of the stem cell products, iPSCs and source cells. This includes information on tumorigenicity, sterility, viability, pluripotency, transcriptomic and genomic profiling, among others. The In-depth Product Characterization module contains two sections: IDCCH Assays and Investigation Initiated Product Analysis. The IDCCH Assays describe stem cell product characterization assays that have been designated by the In-Depth Cell Characterization Hub’s Format Guidance as important for the full characterization of a stem cell product. This section has been subdivided into Microbiology/Sterility Testing, Pyrogenicity/Endotoxin Testing, Virology/Adventitious Agents, Mycoplasma Testing, Identity (Species determination) and Sex (Male/Female), Purity and Heterogeneity, Viability, Senescence, Genetic Stability, Cell Proliferation, Clonal Capacity, Pluripotency/Multipotency, Tumorigenicity, Transcriptome Profiling, Epigenome, Genome Profiling, Proteome, Lipidome and Metabolome, Comprehensive Assessment of Cellular Markers and Potency subsection. Each subsection is designed to contain CDEs to characterize the protocols and materials of these assays, as well as the result characteristics. Although publication of iPSC research currently does not require such stringent characterization of the stem cell products, we provide these CDE subsections as a potential future avenue in improving the reliability and reproducibility of stem cell research.

The Microbiology/Sterility Testing subsection contains CDEs that describe protocols and results from bacteriostasis and fungistasis assays and from rapid microbial detection assays. The Pyrogenicity/Endotoxin Testing subsection contains CDEs that describe the protocol and kinetic turbidimetric, chromogenic, gel-clot results from Limulus Amebocyte Lysate assay. The Virology/Adventitious Agents subsection contains CDEs for retrovirus safety evaluations, *in-vitro*-based susceptibility assays and results of Polymerase Chain Reaction (PCR)-based direct-testing of cells. The Mycoplasma Testing subsection contains CDEs for Mycoplasma culture tests and Mycoplasma PCR test. The Identity (Species determination) and Sex (Male/Female) subsection contains CDEs that detail the verification of the species and sex of the stem cell product, using both PCR-based assays and flow cytometry-based assays. The Purity and Heterogeneity subsection contains CDEs that are used to evaluate the composition profile of cellular preparations and to identify the presence of non-target cell types that may pose risks to subjects receiving a cellular product. CDEs for assays in this subsection include those characterizing RNA-seq, ATAC-seq, flow cytometry and mass cytometry. The Viability subsection CDEs characterize cell viability assays, including Trypan Blue Dye exclusion assay, Acridine Orange and Propidium Iodide (AOPI) staining, Mitochondrial membrane potential-dyes assay and Oxygen consumption and glycolysis activity assays. The Senescence subsection contains CDEs for the Telomere length assay. The Genetic Stability subsection details karyotyping, southern blot analysis and Fluorescence in situ hybridization (FISH) assay CDEs. The Cell Proliferation subsection contains CDEs to describe MTT (3-(4,5-dimethylthiazol-2-yl)-2,5-diphenyltetrazolium bromide) tetrazolium reduction assay for proliferation, Alamar Blue assay for proliferation, Luminescence Adenosine Triphosphate (ATP) assay for proliferation and tests for cell proliferation markers. The Clonal Capacity subsection details the *in*  *vitro* single-cell clonogenic assay. The Pluripotency/Multipotency subsection CDEs detail the Embryoid body formation assay, Specific lineage differentiation results as well as adipogenesis, chondrogenesis and osteogenesis of MSCs. The Tumorigenicity subsection CDEs describe OncoPanel assay, F1CDx assay and MSK-IMPACT assay results. The Transcriptome Profiling CDEs describe the transcriptome protocol and results both from RNA-seq and single molecule-FISH assays. The Epigenome subsection characterizes ATAC-seq assay, ChIP-seq assay and Methylome assay results. The Genome Profiling subsection contains CDEs to describe genome sequencing of the stem cell products. Similarly, the Proteome and Lipidome and Metabolome subsections detail CDEs used to characterize the proteome, lipidome and metabolome using mass spectrometry approaches. The Comprehensive Assessment of Cellular Markers CDEs detail the analysis and results of flow cytometry and electron microscopy. Finally, the Potency subsection CDEs describe the results of cytokine secretion profile analysis, stereotypic morphometric analysis, cell polarization analysis and electrical resistance analysis.

The Investigation Initiated Product Analysis section contains CDEs that can be used to characterize additional stem cell product characterization assays not detailed by the IDCCH. This section is composed of the Assays, Assay metadata and Imaging subsections. The Assays subsection contains CDEs that describe common assay techniques, particularly qPCR. The Assay metadata CDEs contains additional CDEs that describe the data format of the assay results, including data processing and analytical methodologies. Finally, the Imaging section contains CDEs that detail image data collection. These CDEs are adapted to diverse imaging methodologies, including MRI and confocal microscopy. This module also contains space for new CDEs that characterize additional assays to further characterize their stem cell products. As with previous sections, these CDEs will be added as necessary to ensure the full detailed characterization of the stem cell products from published iPSC research projects.

#### Outcomes/Findings module

The Outcomes/Findings module contains CDEs characterizing primary and secondary outcome measures and findings from pre-clinical animal model studies. This module is composed of three sections: Primary Clinical Outcomes, Secondary Clinical Outcomes and Preclinical and In-vitro Findings. These sections are designed to report the key findings from each published project in a standardized CDE format. The Primary Clinical Outcomes and Secondary Clinical Outcomes contain CDEs for outcome measure title, description, type and time frame. As their name implies, these modules are relevant to clinical trials. The formatting of these CDEs is modelled on the formatting of ClinicalTrials.gov outcomes. The Preclinical and In-vitro Findings subsection is designed to accommodate results from pre-clinical and *in-vitro* studies. The CDEs contained within are able to accommodate morphological, functional and pathway results, along with a more generalized experimental finding type.

The modular organization allows for not all sections of the framework to need to be shared across all funded projects, allowing for a more flexible and comprehensive organization of all regenerative medicine trials, regardless of their clinical or pre-clinical status. The modular organization attempts to provide a comprehensive overview of the potential CDEs that can be used to describe a wide variety of stem cell product projects, whose final composition can be adjusted while maintaining a core set of CDEs that will allow for detailed comparisons across studies.

To further illustrate how the multi-modular CDE framework functions for guiding data collection from published iPSC projects, we have included an illustrative example from a publication detailing the generation of an iPSC line, HTN-1_iPSC, from a patient with hypertension (45) (Supplementary Table S2). This example illustrates the typical CDE values collected from an *in-vitro* study, including project information, source cell and iPSC characteristics and manufacturing information and information regarding the experiments performed on the cell line.

### Data visualization and sharing

**Figure 6.**
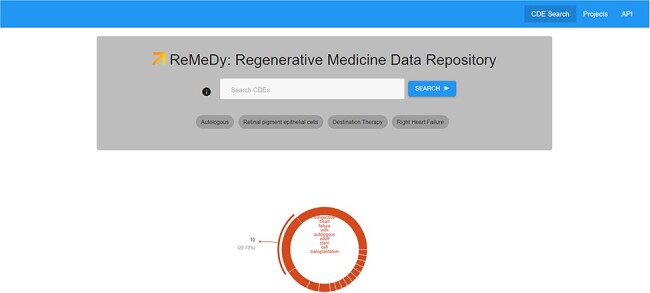
ReMeDy landing page. This image shows the search space, visualization tools, the API platform and Project links functionalities accessible though the landing page.

ReMeDy utilizes the Signature Commons UI to visualize the data and the search results (Figure 6). The highly adaptable search function allows us to search using both keys and values, as well as incorporating Boolean operators to further refine your query. Further, the use of visualization schemas allows us to tailor visualization of libraries and signatures to highlight the most relevant CDEs to enhance the user experience.

The landing page is designed to provide easy access to all the functionalities available through the ReMeDy platform (Figure 6). These include the search space functionality, visualization tools, project links and the API platform. The search functionality is designed to be maximally function by providing advanced search tools with intuitive controls. These controls include Boolean operator functionalities. These Boolean search operators allow the user to exclude word from search by prefixing the query with ‘-’ or ‘!’, to combine searches by prefixing the query with ‘or’ or ‘|’, in addition to the standard combining search for multiple terms that is the equivalent of prefixing the query with ‘and’. The search functionality further allows to search both by CDE name and by CDE value. Additionally, the CDE search is not case sensitive allowing for increased usability of the search functionality. The search space is also customizable to display example search terms or search terms of particular interest to the regenerative medicine community, such as highlighting projects with autologous stem cell products.

The landing page also provides a range of visualization tools to show the data distribution within the ReMeDy database. This functionality is part of the underlying Signature Commons architecture, allowing for creation of bar charts, pie charts, data type counts and key word/value abundance visualizations. The ReMeDy loading page displays a pie chart that show the distribution of clinical patient and pre-clinical animal model and derived cell line subject across the published projects in the database. The ReMeDy landing page also provides access to the API functionality, which allows users to retrieve the data stored within ReMeDy in JSON format. These data can be retrieved both as complete record blocks and as individual records, either by using each record’s unique ID (provided by the UUID) or by restricting the records by CDE name or value. Finally, the ReMeDy landing page provides a link to the Projects page, which lists all the regenerative medicine projects currently stored with the database.

**Figure 7.**
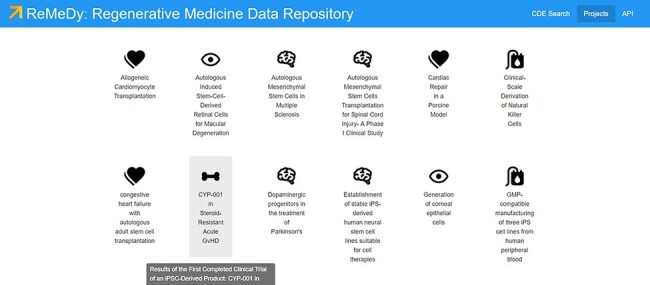
Projects page for ReMeDy, highlighting a subset of published projects currently in the database.

The ReMeDy Projects page list all the projects that have been uploaded to the platform through the API functionality (Figure 7). The current set of projects includes the 51 published iPSC studies. Accessible though the Projects page or through the search functionality, each Projects contains a record of the static CDEs used to describe that project. The project record is designed to store CDEs that comprise the Project, Manufacturing/Production, In-depth Product Characterization, and Outcomes/Findings modules of the multi-modular CDE framework. These CDEs are largely designated as static CDEs, since their values are not expected to change across the course of the study, or across the individual clinical trial patients or pre-clinical animal model subjects. However, in cases where these CDE values are subject to change, such as in the case of autologous stem cell products, multiple projects records can be created and linked to that project, to ensure that all possible relevant iPSC data are captured within the database. The project records additionally serve as links to the individual clinical trial patient and pre-clinical animal model subject records, to ensure full connective of the regenerative medicine data for each project.

**Figure 8.**
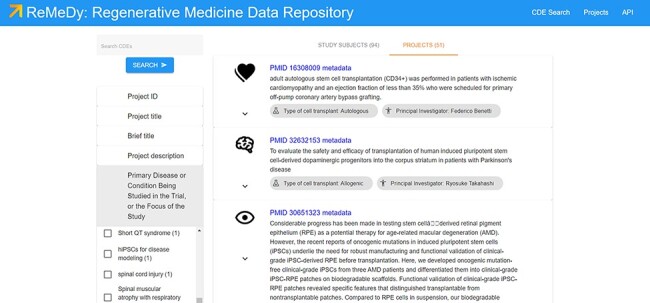
Search results page, displaying all the data currently in the ReMeDy database and highlighting the implemented filtering functionality.

The search functionality provides access to the individual and grouped research subject records (Figure 8), in addition to the project records also accessible through the Projects page. The search functionality works for both records types (library and signature) automatically. This enhances the end user’s ability to quickly retrieve all relevant data. Although searching for a specific CDE name or value allows for display of specific records, a blank search returns all the data currently in the ReMeDy database. This total includes, 94 signatures, comprising of clinical patients, animal models and cell lines, and 51 libraries, containing information on iPSCs and their derivatives used in the 51 published studies. Selecting one of the records allows for all the CDEs contained to be accessed. The project records highlight the high degree of customizability that is available through the underlying Signature Commons architecture, using the visualization schema. Here, we highlight two important aspects of iPSC research: the type of iPSC product under investigation (allogeneic or autologous) and the lead PI of each project. This allows each project to be individually tailored to highlight the most relevant CDE values and improve the engagement and integrated usability of the stored data to the end user. To further improve usability, we have implemented filtering utilities for all the CDEs in the multi-modular CDE framework (Figure 7). Not only does this filtering utility allow the user to further refine their search query, but the count associated with each value provides a quick and efficient way to obtain statistics on the data composition of the ReMeDy platform.

**Figure 9.**
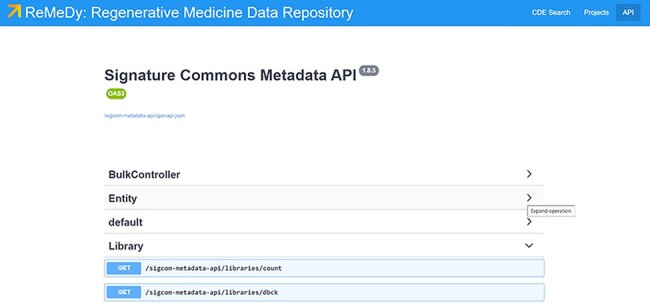
ReMeDy API. The ReMeDy API is based on the underlying Signature Commons architecture, which provides extensive documentation of the API functionalities.

The ReMeDy API allows for access to both project and study subject records (Figure 9). To facilitate future research and collaboration, ReMeDy allows researchers to download the database data direction through the API. The benefits of this feature are the compartmentalization of the data, allowing the desired data to be restricted by data type (resources, libraries and signatures). The API is annotated using Swagger 2.0 JSON implementation, and all RESTful endpoints return structured JSON. Multiple endpoints allow searching content by any of its curated metadata annotations as well as requesting different slices of metadata associated with any of the subcategories of CDE modules. Further, individual data records can be retrieved using the UUID. Since the UUIDs are also visible in the search results, this allows for flexible, on-demand data retrieval. The underlying Signature Commons API provides ample documentation of the available API commons, to increase the usability and user-friendliness of the API platform. The aim is to promote easy access to the data to the end-user in order to further foster community sharing and collaboration based around the regenerative medicine data stored within ReMeDy and help drive advancements is regenerative medicine.

**Figure 10.**
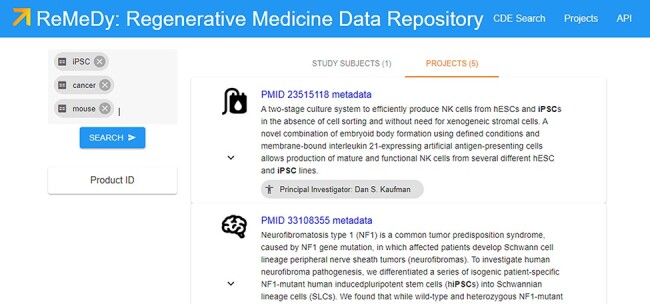
Demonstration of a search query used to collect cohorts for further analysis.

To illustrate how the ReMeDy database can be used for further study, we provide an example search query that collects a cohort of iPSC data and publications that can then be used for additional analysis. Querying the iPSC data in the ReMeDy database can be accomplished using the implemented search function and filtering schemas. For example, to find projects in ReMeDy that are using iPSCs, working on mice and are studying cancer, the search terms ‘iPSC’, ‘mouse’, and ‘cancer’ can be iteratively entered into the search bar (Figure 10). This search returns five projects. For preliminary statistical analyses, the CDE values across these projects are summed up in the filtering schemas. To obtain the CDE data for these projects programmatically, the following command can be implemented: curl -X POST "http://localhost/sigcom-metadata-api/libraries/find" -H "accept: application/json" -H "Content-Type: application/json" -d "{\"filter\":{\"meta.Project.General%20Information.Project%20description\":[\"mouse\"],\"meta.Project.General%20Information.Primary%20Disease%20or%20Condition%20Being%20Studied%20in%20the%20Trial,%20or%20the%20Focus%20of%20the%20Study\":[\"cancer\"]},\"contentRange\":true}". Having identified the study cohort, we can begin to ask scientific questions about the similarities or differences across these studies. For example, from the described cohort, we can identify that two of them focus on characterization of stem cell products using CD8 expression, while other studies focus on CD34 and CD45 or SOX10. Thus the ReMeDy platform has the potential to drive knowledge generation by providing researchers with an easy to access information on iPSC characteristics, clinical outcomes and research finding, and research subject level data.

## Discussion

The expanding field of regenerative medicine required the creating of a flexible and agile repository for stem cell data aggregation, storage, visualization and sharing; therefore, we launched ReMeDy platform. ReMeDy is an organized repository that captures iPSC-research project information in standardized and effortless to visualize format. The advantage of our architecture is the utilization of an interactive database with JSON formatting to import and store data, which allows us to incorporate large data sets in a schema-less nature while simultaneously employing validators to ensure stringent quality control measures. We further improve the long-term usability and viability of our platform by providing the researchers with a convenient tool to import their own data using simple tsv file formats, allowing them to bypass more complicated computational stages of JSON generation. The platform promotes easy accessibility to regenerative medicine projects to facilitate data sharing and collaboration within the field. The data upload interface includes the generation of a custom metadata validator, necessary for validation against ontologies and identification of required elements for ingestion, which allows us to select the most informative stem cells CDEs. This list of essential CDEs not only includes primary parameters to characterize cell products but also allows for standardized cross-discipline and cross-studies comparison. In addition, ReMeDy’s landing page provides access to all regenerative medicine projects, currently stored within the database.

We tested the platform by uploading 51 PubMed published projects, including *in vitro*, pre-clinical and clinical studies, in a systemic organized manner, using a flexible framework of core harmonized CDE. The assessment revealed that the simplicity and flexibility of ReMeDy allows for the storage and visualization of metadata for any project, as long as a standardized CDE template is generated. Future plans for ReMeDy include on-going updates to the platform, while simultaneously improving its functionality and serviceability for the expanding number of regenerative medicine projects. We believe that ReMeDy is a repository to fulfil such demands.

## Future directions

Future plans for the ReMeDy database include making the platform available for other research groups. The simplicity of submitting spreadsheets of CDEs, which can easily be transformed to tab separated value (.tsv) file format, allows for the crowdsourcing of the platform. In addition, our efforts include establishing an automated pipeline for uploading the CDE templates, which can be utilized by researchers. We plan to transform the upload interface into a publically facing webpage. To ensure the quality of data submission, we will implement a login interface that will allow researchers to create submission accounts. This will enable them to submit CDE templates in the format of our multi-modular CDE framework. Advantages of crowdsourcing will include not only the ensuring that the iPSC project data stays up to date, but further encouraging engagement with the platform, thus further driving the development of potential collaborations and promoting advances in regenerative medicine.

## Supplementary Material

baab038_Supp

## References

[1] Finkelstein J. , Parvanova I. and Zhang F. (2020) Informatics approaches for harmonized intelligent integration of stem cell research. *Stem Cells Cloning*, 13, 1–20. 32099411 10.2147/SCCAA.S237361 PMC6996484

[2] Borziak K. , Qi T., Evangelista J.E. et al. (2020) Towards intelligent integration and sharing of stem cell research data. *Stud Health Technol. Inform.*, 272, 334–337. 32604670 10.3233/SHTI200563

[3] Keenan A.B. , Jenkins S.L., Jagodnik K.M. et al. (2018) The library of integrated network-based cellular signatures NIH program: system-level cataloging of human cells response to perturbations. *Cell Syst.*, 6, 13–24. 29199020 10.1016/j.cels.2017.11.001 PMC5799026

[4] Doi D. , Magotani H., Kikuchi T. et al. (2020) Pre-clinical study of induced pluripotent stem cell-derived dopaminergic progenitor cells for Parkinson’s disease. *Nat. Commun.*, 11, 3369. 10.1038/s41467-020-17165-w PMC7338530 32632153

[5] Sharma R. , Khristov V., Rising A. et al. (2019) Clinical-grade stem cell-derived retinal pigment epithelium patch rescues retinal degeneration in rodents and pigs. *Sci. Transl. Med.*, 11, eaat5580. 10.1126/scitranslmed.aat5580 PMC8784963 30651323

[6] Bloor A.J.C. , Patel A., Griffin J.E. et al. (2020) Production, safety and efficacy of iPSC-derived mesenchymal stromal cells in acute steroid-resistant graft versus host disease: a phase I, multicenter, open-label, dose-escalation study. *Nat. Med.*, 26, 1720–1725. 32929265 10.1038/s41591-020-1050-x

[7] Yoshida S. , Miyagawa S., Toyofuku T. et al. (2020) Syngeneic mesenchymal stem cells reduce immune rejection after induced pluripotent stem cell-derived allogeneic cardiomyocyte transplantation. *Sci. Rep.*, 10, 4593. 10.1038/s41598-020-58126-z PMC7067786 32165680

[8] Llufriu S. , Sepúlveda M., Blanco Y. et al. (2014) Randomized placebo-controlled phase II trial of autologous mesenchymal stem cells in multiple sclerosis. *PLoS One*, 9, e113936. 10.1371/journal.pone.0113936 PMC4250058 25436769

[9] Ishida M. , Miyagawa S., Saito A. et al. (2019) Transplantation of human-induced pluripotent stem cell-derived cardiomyocytes is superior to somatic stem cell therapy for restoring cardiac function and oxygen consumption in a porcine model of myocardial infarction. *Transplantation*, 103, 291–298. 30119058 10.1097/TP.0000000000002384 PMC6365242

[10] Tang H. , Sha H., Sun H. et al. (2013) Tracking induced pluripotent stem cells-derived neural stem cells in the central nervous system of rats and monkeys. *Cell Reprogram*, 15, 435–442. 24020696 10.1089/cell.2012.0081 PMC3787483

[11] Kagia A. , Tzetis M., Kanavakis E. et al. (2019) Therapeutic effects of mesenchymal stem cells derived from bone marrow, umbilical cord blood, and pluripotent stem cells in a mouse model of chemically induced inflammatory bowel disease. *Inflammation*, 42, 1730–1740. 31227956 10.1007/s10753-019-01033-x

[12] Palma-Tortosa S. , Tornero D., Grønning Hansen M. et al. (2020) Activity in grafted human iPS cell-derived cortical neurons integrated in stroke-injured rat brain regulates motor behavior. *Proc. Natl. Acad. Sci. U**S**A.*, 117, 9094–9100. 32253308 10.1073/pnas.2000690117 PMC7183146

[13] Khan M.A. , Alanazi F., Ahmed H.A. et al. (2019) iPSC-derived MSC therapy induces immune tolerance and supports long-term graft survival in mouse orthotopic tracheal transplants. *Stem Cell Res. Ther.*, 10, 290. 10.1186/s13287-019-1397-4 PMC6757436 31547869

[14] Tabei R. , Kawaguchi S., Kanazawa H. et al. (2019) Development of a transplant injection device for optimal distribution and retention of human induced pluripotent stem cell‒derived cardiomyocytes. *J. Heart Lung Transplant.*, 38, 203–214. 30691596 10.1016/j.healun.2018.11.002

[15] Ra J.C. , Shin I.S., Kim S.H. et al. (2011) Safety of intravenous infusion of human adipose tissue-derived mesenchymal stem cells in animals and humans. *Stem Cells Dev.*, 20, 1297–1308. 21303266 10.1089/scd.2010.0466

[16] Satti H.S. , Waheed A., Ahmed P. et al. (2016) Autologous mesenchymal stromal cell transplantation for spinal cord injury: a Phase I pilot study. *Cytotherapy*, 18, 518–522. 26971680 10.1016/j.jcyt.2016.01.004

[17] Happle C. , Lachmann N., Škuljec J. et al. (2014) Pulmonary transplantation of macrophage progenitors as effective and long-lasting therapy for hereditary pulmonary alveolar proteinosis. *Sci. Transl. Med.*, 6, 250. 10.1126/scitranslmed.3009750 25143363

[18] Knorr D.A. , Ni Z., Hermanson D. et al. (2013) Clinical-scale derivation of natural killer cells from human pluripotent stem cells for cancer therapy. *Stem Cells Transl. Med.*, 2, 274–283. 23515118 10.5966/sctm.2012-0084 PMC3659832

[19] Yau T.M. , Pagani F.D., Mancini D.M. et al. (2019) Intramyocardial injection of mesenchymal precursor cells and successful temporary weaning from left ventricular assist device support in patients with advanced heart failure: a randomized clinical trial. *JAMA*, 321, 1176–1186. 30912838 10.1001/jama.2019.2341 PMC6439694

[20] Akabayashi A. , Nakazawa E., Jecker N.S. et al. (2019) The world’s first clinical trial for an aplastic anemia patient with thrombocytopenia administering platelets generated from autologous iPS cells. *Int. J. Hematol.*, 109, 239–240. 30535854 10.1007/s12185-018-02565-y

[21] Forotti G. , Nizzardo M., Bucchia M. et al. (2019) CSF transplantation of a specific iPSC-derived neural stem cell subpopulation ameliorates the disease phenotype in a mouse model of spinal muscular atrophy with respiratory distress type 1. *Exp. Neurol.*, 321, 113041. 10.1016/j.expneurol.2019.113041 31445043

[22] Patel A.N. , Geffner L., Vina R.F. et al. (2005) Surgical treatment for congestive heart failure with autologous adult stem cell transplantation: a prospective randomized study. *J. Thorac. Cardiovasc. Surg.*, 130, 1631–1638. 16308009 10.1016/j.jtcvs.2005.07.056

[23] Nizzardo M. , Simone C., Rizzo F. et al. (2014) Minimally invasive transplantation of iPSC-derived ALDHhiSSCloVLA4+ neural stem cells effectively improves the phenotype of an amyotrophic lateral sclerosis model. *Hum. Mol. Genet.*, 23, 342–354. 24006477 10.1093/hmg/ddt425 PMC3869354

[24] López-Serrano C. , Torres-Espín A., Hernández J. et al. (2016) Effects of the post-spinal cord injury microenvironment on the differentiation capacity of human neural stem cells derived from induced pluripotent stem cells. *Cell Transplant.*, 25, 1833–1852. 27075820 10.3727/096368916X691312

[25] Liu Y. , Zheng Y., Li S. et al. (2017) Human neural progenitors derived from integration-free iPSCs for SCI therapy. *Stem. Cell Res.*, 19, 55–64. 28073086 10.1016/j.scr.2017.01.004 PMC5629634

[26] Guo F. , Sun Y., Wang X. et al. (2019) Patient-specific and gene-corrected induced pluripotent stem cell-derived cardiomyocytes elucidate single-cell phenotype of short QT syndrome. *Circ. Res.*, 124, 66–78. 30582453 10.1161/CIRCRESAHA.118.313518

[27] Haase A. , Glienke W., Engels L. et al. (2019) GMP-compatible manufacturing of three iPS cell lines from human peripheral blood. *Stem Cell Res.*, 35, 101394. 10.1016/j.scr.2019.101394 30772682

[28] Palma-Tortosa S. , Tornero D., Grønning H.M. et al. (2020) Activity in grafted human iPS cell-derived cortical neurons integrated in stroke-injured rat brain regulates motor behavior. *Proc. Natl. Acad. Sci. U**S**A*, 117, 9094–9100. 32253308 10.1073/pnas.2000690117 PMC7183146

[29] Kimura T. , Fukushima S., Okada E. et al. (2020) Induced pluripotent stem cell-derived myeloid cells expressing OX40 ligand amplify antigen-specific T cells in advanced melanoma. *Pigment Cell Melanoma Res.*, 33, 744–755. 32353897 10.1111/pcmr.12887

[30] Abulaiti M. , Yalikun Y., Murata K. et al. (2020) Establishment of a heart-on-a-chip microdevice based on human iPS cells for the evaluation of human heart tissue function. *Sci Rep.*, 10, 19201. 10.1038/s41598-020-76062-w PMC7645446 33154509

[31] Lee S.J. , Kim J.H., Kang K.W. et al. (2020) Generation of normal induced pluripotent stem cell line KUMCi002-A from bone marrow CD34+ cells of patient with multiple myeloma disease having 13q deletion and IGH translocation. *Stem Cell Res.*, 49, 102030. 10.1016/j.scr.2020.102030 33142253

[32] Fukushima H. , Yoshioka M., Kawatou M. et al. (2020) Specific induction and long-term maintenance of high purity ventricular cardiomyocytes from human induced pluripotent stem cells. *PLoS One*, 15, e0241287. 10.1371/journal.pone.0241287 PMC7605685 33137106

[33] Luo J. , Lin Y., Shi X. et al. (2020) Xenogeneic-free generation of vascular smooth muscle cells from human induced pluripotent stem cells for vascular tissue engineering. *Acta Biomater.*, 7061, 30640–30641. 10.1016/j.actbio.2020.10.042 PMC8168373 33130306

[34] Mo J. , Anastasaki C., Chen Z. et al. (2020) Humanized neurofibroma model from induced pluripotent stem cells delineates tumor pathogenesis and developmental origins. *J. Clin. Invest.*, 131, e139807. 10.1172/JCI139807 PMC7773354 33108355

[35] Ozay E.I. , Vijayaraghavan J., Gonzalez-Perez G. et al. (2019) Cymerus™ iPSC-MSCs significantly prolong survival in a pre-clinical, humanized mouse model of Graft-vs-host disease. *Stem Cell Res.*, 35, 101401. 10.1016/j.scr.2019.101401 PMC6544140 30738321

[36] Mandai M. , Watanabe A., Kurimoto Y. et al. (2017) Autologous induced stem-cell-derived retinal cells for macular degeneration. *N. Engl. J. Med.*, 376, 1038–1046. 28296613 10.1056/NEJMoa1608368

[37] Rosati J. , Ferrari D., Altieri F. et al. (2017) Establishment of stable iPS-derived human neural stem cell lines suitable for cell therapies. *Cell Death Dis.*, 9, 937. 10.1038/s41419-018-0990-2 PMC6141489 30224709

[38] Minagawa A. , Yoshikawa T., Yasukawa M. et al. (2017) Enhancing T cell receptor stability in rejuvenated iPSC-Derived T cells improves their use in cancer immunotherapy. *Cell Stem Cell*, 23, 850–858. 10.1016/j.stem.2018.10.005 30449714

[39] Tsuchiya N. , Zhang R., Iwama T. et al. (2019) Type I interferon delivery by iPSC-derived myeloid cells elicits antitumor immunity via XCR1^+^ dendritic cells. *Cell Rep.*, 29, 162–175. 31577946 10.1016/j.celrep.2019.08.086

[40] Oki K. , Tatarishvili J., Wood J. et al. (2012) Human-induced pluripotent stem cells form functional neurons and improve recovery after grafting in stroke-damaged brain. *Stem Cells*, 30, 1120–1133. 22495829 10.1002/stem.1104

[41] Ye L. , Chang Y.H., Xiong Q. et al. (2014) Cardiac repair in a porcine model of acute myocardial infarction with human induced pluripotent stem cell-derived cardiovascular cells. *Cell Stem Cell*, 15, 750–761. 25479750 10.1016/j.stem.2014.11.009 PMC4275050

[42] Li Y. , Tsai Y.T., Hsu C.W. et al. (2012) Long-term safety and efficacy of human-induced pluripotent stem cell (iPS) grafts in a preclinical model of retinitis pigmentosa. *Mol. Med.*, 18, 1312–1319. 22895806 10.2119/molmed.2012.00242 PMC3521789

[43] Biel N.M. , Santostefano K.E., DiVita B.B. et al. (2015) Vascular smooth muscle cells from hypertensive patient-derived induced pluripotent stem cells to advance hypertension pharmacogenomics. *Stem Cells Transl. Med.*, 4, 1380–1390. 26494780 10.5966/sctm.2015-0126 PMC4675511

[44] Cao L. , McDonnell A., Nitzsche A. et al. (2016) Pharmacological reversal of a pain phenotype in iPSC-derived sensory neurons and patients with inherited erythromelalgia. *Sci. Transl. Med.*, 8, 335–356. 10.1126/scitranslmed.aad7653 27099175

[45] Geng Z. , Walsh P.J., Truong V. et al. (2017) Generation of retinal pigmented epithelium from iPSCs derived from the conjunctiva of donors with and without age related macular degeneration. *PLoS One*, 12, e0173575. 10.1371/journal.pone.0173575 PMC5345835 28282420

[46] Cha Y. , Han M.J., Cha H.J. et al. (2017) Metabolic control of primed human pluripotent stem cell fate and function by the miR-200c-SIRT2 axis. *Nat. Cell Biol.*, 19, 445–456. 28436968 10.1038/ncb3517 PMC5545746

[47] Kohara H. , Utsugisawa T., Sakamoto C. et al. (2019) KLF1 mutation E325K induces cell cycle arrest in erythroid cells differentiated from congenital dyserythropoietic anemia patient-specific induced pluripotent stem cells. *Exp. Hematol.*, 73, 25–37. 30876823 10.1016/j.exphem.2019.03.001

[48] Isogai S. , Yamamoto N., Hiramatsu N. et al. (2019) Preparation of induced pluripotent stem cells using human peripheral blood monocytes. *Cell Reprogram*, 20, 347–355. 10.1089/cell.2018.0024 PMC6302673 31107605

[49] Sun X. , Song J., Huang H. et al. (2018) Modeling hallmark pathology using motor neurons derived from the family and sporadic amyotrophic lateral sclerosis patient-specific iPS cells. *Stem Cell Res. Ther.*, 9, 315. 10.1186/s13287-018-1048-1 PMC6238404 30442180

[50] Bossolasco P. , Sassone F., Gumina V. et al. (2018) Motor neuron differentiation of iPSCs obtained from peripheral blood of a mutant TARDBP ALS patient. *Stem Cell Res.*, 30, 61–68. 29800782 10.1016/j.scr.2018.05.009

[51] Peng Y. , Liou B., Inskeep V. et al. (2019) Intravenous infusion of iPSC-derived neural precursor cells increases acid β-glucosidase function in the brain and lessens the neuronopathic phenotype in a mouse model of Gaucher disease. *Hum. Mol. Genet.*, 28, 3406–3421. 31373366 10.1093/hmg/ddz184 PMC6891072

[52] Yamamoto Y. , Gotoh S., Korogi Y. et al. (2017) Long-term expansion of alveolar stem cells derived from human iPS cells in organoids. *Nat. Methods*, 14, 1097–1106. 28967890 10.1038/nmeth.4448

[53] Li Z. , Duan H., Li W. et al. (2019) Rapid differentiation of multi-zone ocular cells from human induced pluripotent stem cells and generation of corneal epithelial and endothelial cells. *Stem Cells Dev.*, 28, 454–463. 30712489 10.1089/scd.2018.0176

[54] Hayashi R. , Ishikawa Y., Ito M. et al. (2012) Generation of corneal epithelial cells from induced pluripotent stem cells derived from human dermal fibroblast and corneal limbal epithelium. *PLoS One*, 7, e45435. 10.1371/journal.pone.0045435 PMC3454439 23029008

[55] Sakurai K. , Kurtz A., Stacey G. et al. (2016) First proposal of minimum information about a cellular assay for regenerative medicine. *Stem Cells Transl. Med.*, 5, 1345–1361. 27405781 10.5966/sctm.2015-0393 PMC5031183

