# Supplementary material for: ReMeDy: a platform for integrating and sharing published stem cell research data with a focus on iPSC trials
Source: Database (Oxford). 2021 Jun 22;2021:baab038. doi: 10.1093/database/baab038 (PMC8218701; doi:10.1093/database/baab038)
Supplement: baab038_Supp [file baab038_supp.zip › suppl_data/Supplementary Table 1.docx]

| Publication title | PubMed ID | Reference |
| --- | --- | --- |
| Pre-clinical study of induced pluripotent stem cell-derived dopaminergic progenitor cells for Parkinson's disease | 32632153 | (4) |
| Clinical-grade stem cell-derived retinal pigment epithelium patch rescues retinal degeneration in rodents and pigs | 30651323 | (5) |
| Production, safety and efficacy of iPSC-derived mesenchymal stromal cells in acute steroid-resistant graft versus host disease: a phase I, multicenter, open-label, dose-escalation study | 32929265 | (6) |
| Syngeneic Mesenchymal Stem Cells Reduce Immune Rejection After Induced Pluripotent Stem Cell-Derived Allogeneic Cardiomyocyte Transplantation | 32165680 | (7) |
| Randomized placebo-controlled phase II trial of autologous mesenchymal stem cells in multiple sclerosis | 25436769 | (8) |
| Transplantation of Human-induced Pluripotent Stem Cell-derived Cardiomyocytes Is Superior to Somatic Stem Cell Therapy for Restoring Cardiac Function and Oxygen Consumption in a Porcine Model of Myocardial Infarction | 30119058 | (9) |
| Tracking induced pluripotent stem cells-derived neural stem cells in the central nervous system of rats and monkeys | 24020696 | (10) |
| Therapeutic Effects of Mesenchymal Stem Cells Derived From Bone Marrow, Umbilical Cord Blood, and Pluripotent Stem Cells in a Mouse Model of Chemically Induced Inflammatory Bowel Disease | 31227956 | (11) |
| Activity in grafted human iPS cell-derived cortical neurons integrated in stroke-injured rat brain regulates motor behavior | 32253308 | (12) |
| iPSC-derived MSC therapy induces immune tolerance and supports long-term graft survival in mouse orthotopic tracheal transplants | 31547869 | (13) |
| Development of a transplant injection device for optimal distribution and retention of human induced pluripotent stem cell‒derived cardiomyocytes | 30691596 | (14) |
| Safety of intravenous infusion of human adipose tissue-derived mesenchymal stem cells in animals and humans | 21303266 | (15) |
| Autologous mesenchymal stromal cell transplantation for spinal cord injury: A Phase I pilot study | 26971680 | (16) |
| Pulmonary transplantation of macrophage progenitors as effective and long-lasting therapy for hereditary pulmonary alveolar proteinosis | 25143363 | (17) |
| Clinical-scale derivation of natural killer cells from human pluripotent stem cells for cancer therapy | 23515118 | (18) |
| Intramyocardial Injection of Mesenchymal Precursor Cells and Successful Temporary Weaning From Left Ventricular Assist Device Support in Patients With Advanced Heart Failure: A Randomized Clinical Trial | 30912838 | (19) |
| The world’s first clinical trial for an aplastic anemia patient with thrombocytopenia administering platelets generated from autologous iPS cells | 30535854 | (20) |
| CSF transplantation of a specific iPSC-derived neural stem cell subpopulation ameliorates the disease phenotype in a mouse model of spinal muscular atrophy with respiratory distress type 1 | 31445043 | (21) |
| Surgical treatment for congestive heart failure with autologous adult stem cell transplantation: A prospective randomized study | 16308009 | (22) |
| Minimally invasive transplantation of iPSC-derived ALDHhiSSCloVLA4+ neural stem cells effectively improves the phenotype of an amyotrophic lateral sclerosis model | 24006477 | (23) |
| Effects of the Post-Spinal Cord Injury Microenvironment on the Differentiation Capacity of Human Neural Stem Cells Derived from Induced Pluripotent Stem Cells | 27075820 | (24) |
| Human neural progenitors derived from integration-free iPSCs for SCI therapy | 28073086 | (25) |
| Patient-Specific and Gene-Corrected Induced Pluripotent Stem Cell-Derived Cardiomyocytes Elucidate Single-Cell Phenotype of Short QT Syndrome | 30582453 | (26) |
| GMP-compatible manufacturing of three iPS cell lines from human peripheral blood | 30772682 | (27) |
| Isolated allogeneic bone marrow-derived mesenchymal cells engraft and stimulate growth in children with osteogenesis imperfecta: Implications for cell therapy of bone | 12084934 | (28) |
| Induced pluripotent stem cell‐derived myeloid cells expressing OX40 ligand amplify antigen‐specific T cells in advanced melanoma | 32353897 | (29) |
| Establishment of a heart-on-a-chip microdevice based on human iPS cells for the evaluation of human heart tissue function | 33154509 | (30) |
| Generation of normal induced pluripotent stem cell line KUMCi002-A from bone marrow CD34+ cells of patient with multiple myeloma disease having 13q deletion and IGH translocatione | 33142253 | (31) |
| Specific induction and long-term maintenance of high purity ventricular cardiomyocytes from human induced pluripotent stem cells | 33137106 | (32) |
| Xenogeneic-free generation of vascular smooth muscle cells from human induced pluripotent stem cells for vascular tissue engineering | 33130306 | (33) |
| Humanized neurofibroma model from induced pluripotent stem cells delineates tumor pathogenesis and developmental origins | 33108355 | (34) |
| Cymerus™ iPSC-MSCs significantly prolong survival in a pre-clinical, humanized mouse model of Graft-vs-host disease | 30738321 | (35) |
| Autologous Induced Stem-Cell–Derived Retinal Cells for Macular Degeneration | 28296613 | (36) |
| Establishment of stable iPS-derived human neural stem cell lines suitable for cell therapies | 30224709 | (37) |
| Enhancing T Cell Receptor Stability in Rejuvenated iPSC-Derived T Cells Improves Their Use in Cancer Immunotherapy | 30449714 | (38) |
| Type I Interferon Delivery by iPSC-Derived Myeloid Cells Elicits Antitumor Immunity via XCR1+ Dendritic Cells | 31577946 | (39) |
| Human‐Induced Pluripotent Stem Cells form Functional Neurons and Improve Recovery After Grafting in Stroke‐Damaged Brain | 22495829 | (40) |
| Cardiac Repair in a Porcine Model of Acute Myocardial Infarction with Human Induced Pluripotent Stem Cell-Derived Cardiovascular Cells | 25479750 | (41) |
| Long-term Safety and Efficacy of Human-Induced Pluripotent Stem Cell (iPS) Grafts in a Preclinical Model of Retinitis Pigmentosa | 22895806 | (42) |
| Vascular Smooth Muscle Cells From Hypertensive Patient-Derived Induced Pluripotent Stem Cells to Advance Hypertension Pharmacogenomics | 26494780 | (43) |
| Pharmacological reversal of a pain phenotype in iPSC-derived sensory neurons and patients with inherited erythromelalgia | 27099175 | (44) |
| Generation of retinal pigmented epithelium from iPSCs derived from the conjunctiva of donors with and without age related macular degeneration | 28282420 | (45) |
| Metabolic control of primed human pluripotent stem cell fate and function by the miR-200c–SIRT2 axis | 28436968 | (46) |
| KLF1 mutation E325K induces cell cycle arrest in erythroid cells differentiated from congenital dyserythropoietic anemia patient-specific induced pluripotent stem cells | 30876823 | (47) |
| Preparation of Induced Pluripotent Stem Cells Using Human Peripheral Blood Monocytes | 31107605 | (48) |
| Modeling hallmark pathology using motor neurons derived from the family and sporadic amyotrophic lateral sclerosis patient-specific iPS cells | 30442180 | (49) |
| Motor neuron differentiation of iPSCs obtained from peripheral blood of a mutant TARDBP ALS patient | 29800782 | (50) |
| Intravenous infusion of iPSC-derived neural precursor cells increases acid β-glucosidase function in the brain and lessens the neuronopathic phenotype in a mouse model of Gaucher disease | 31373366 | (51) |
| Long-term expansion of alveolar stem cells derived from human iPS cells in organoids | 28967890 | (52) |
| Rapid Differentiation of Multi-Zone Ocular Cells from Human Induced Pluripotent Stem Cells and Generation of Corneal Epithelial and Endothelial Cells | 30712489 | (53) |
| Generation of corneal epithelial cells from induced pluripotent stem cells derived from human dermal fibroblast and corneal limbal epithelium | 23029008 | (54) |
